# Molecular control of HIV-1 postintegration latency: implications for the development of new therapeutic strategies

**DOI:** 10.1186/1742-4690-6-111

**Published:** 2009-12-04

**Authors:** Laurence Colin, Carine Van Lint

**Affiliations:** 1 Laboratory of Molecular Virology, Institut de Biologie et de Médecine Moléculaires (IBMM), Université Libre de Bruxelles (ULB), 6041 Gosselies, Belgium

## Abstract

The persistence of HIV-1 latent reservoirs represents a major barrier to virus eradication in infected patients under HAART since interruption of the treatment inevitably leads to a rebound of plasma viremia. Latency establishes early after infection notably (but not only) in resting memory CD4^+ ^T cells and involves numerous host and viral trans-acting proteins, as well as processes such as transcriptional interference, RNA silencing, epigenetic modifications and chromatin organization. In order to eliminate latent reservoirs, new strategies are envisaged and consist of reactivating HIV-1 transcription in latently-infected cells, while maintaining HAART in order to prevent *de novo *infection. The difficulty lies in the fact that a single residual latently-infected cell can in theory rekindle the infection. Here, we review our current understanding of the molecular mechanisms involved in the establishment and maintenance of HIV-1 latency and in the transcriptional reactivation from latency. We highlight the potential of new therapeutic strategies based on this understanding of latency. Combinations of various compounds used simultaneously allow for the targeting of transcriptional repression at multiple levels and can facilitate the escape from latency and the clearance of viral reservoirs. We describe the current advantages and limitations of immune T-cell activators, inducers of the NF-κB signaling pathway, and inhibitors of deacetylases and histone- and DNA- methyltransferases, used alone or in combinations. While a solution will not be achieved by tomorrow, the battle against HIV-1 latent reservoirs is well- underway.

## 

A quarter of a century after the discovery of HIV-1, we are still unable to eradicate the virus from infected patients. Highly active antiretroviral therapy (HAART) consists of combinations of antiretroviral therapeutics targeting different steps of the virus life cycle (e.g. entry, reverse transcription, integration and maturation) used simultaneously to reduce the risk of viral replication and the development of drug resistance conferred by the emergence of mutant strains [1-3]. HAART results in a four-phase decay of viremia [4-7]: (1) an initial rapid loss of virus due to the clearance of infected activated CD4^+ ^T cells, which have a very short half-life and survive for about one day because of viral cytopathic effects or host cytolytic effector mechanisms; (2) a slower phase of viral decay owing to the clearance of several cell populations with a half-life of one to four weeks, such as infected macrophages, partially activated CD4^+ ^T cells and follicular dendritic cells (FDCs); (3) a third phase of decay corresponding to cells with a half-life of approximately 39 weeks; and (4) a constant phase with no appreciable decline, caused (at least partially) by the activation of resting memory CD4^+ ^T cells. During the fourth phase, HIV-1 plasma viremia normally ranges from 1 to 5 copies of viral RNA/mL as detected by extremely sensitive RT-PCR assays [8-10]. Despite the observation that prolonged HAART treatment is associated with many metabolic disorders and toxicities [11,12], the prospect of lifelong treatment is today a necessary evil because interrupting HAART leads to a rapid viral rebound, attributable to the persistence of latently-infected cellular reservoirs notably in resting memory CD4^+ ^T cells [13-15] and probably in other cell populations [16-18]. Viral reservoirs include cell types or anatomical sites where a replication-competent form of the virus persists with more stable kinetics than the main pool of actively replicating virus [5,19]. Because they express no viral protein, latently-infected reservoir cells are immunologically indistinguishable from uninfected cells and are insensitive to immune clearance and HAART.

The persistence of transcriptionally silent but replication-competent HIV-1 reservoirs in HAART-treated infected individuals represents a major hurdle to virus eradication. To address this problem, a first approach has consisted of strengthening HAART. This intensification strategy relied on the administration of additional viral inhibitors in association with HAART. Despite their cytotoxicity, candidate drugs have included hydroxyurea and cyclophosphamide. Hydroxyurea inhibits the cellular enzyme ribonucleotide reductase, thereby decreasing intracellular deoxyribonucleotide pools and indirectly impeding viral reverse transcriptase activity [20,21]. Cyclophosphamide is an alkylating agent that results in cytoreduction and cell growth arrest, and is used to treat various types of cancers and immune diseases. However, these compounds have not been found to decrease the latently-infected reservoirs in HIV-infected patients [22,23].

The source of the observed persistent steady-state viremia in HAART-treated patients has been attributed, on the one hand, to a non-fully suppressive HAART following poor drug penetration in anatomical sanctuaries such as the central nervous system (CNS)[24,25]; and, on the other hand, to the release of virus due to the reactivation of latently-infected resting CD4^+ ^T cells (or other cellular reservoirs) despite fully suppressive therapy. Several groups have proposed the existence of a residual continuous HIV-1 replication, which could constantly replenish the latent pool. This proposition was based on the observation of so-called 2-LTR cirle forms of the provirus, whose half-life should be less than one day reflecting recent rounds of infection, in the plasma of HAART-treated patients [26-29]. However, other groups have found evidence that 2-LTR circles are actually stable and that their apparent decline reflects dilution following cell division [30,31]. In addition, intensified HAART would have prevented this low-level viral replication, and therefore would have accelerated the decay of the latent pool; but such results weren't observed [22,23]. Furthermore, several studies including mathematical modelings of infected cell turnover [5,6,32] and other experimental data [33] suggested that persistent viremia is likely due to the intrinsic stability and reactivation rate of the latently-infected CD4^+ ^T cell reservoir. Given that memory T cells provide long-term immunological memory for decades, their mean half-life can reach 44.2 months. Based upon previous estimation of 10^6 ^cells as the latent reservoir size, Siliciano and colleagues calculated that an average of 60 years of uninterrupted HAART would be necessary to eradicate this latent reservoir [34]. The same group has also recently shown that a source other than circulating resting CD4^+ ^T cells contributes to residual viremia and viral persistence, underscoring the importance of extending HIV-1 reservoir eradication studies to other cell types [35]. Together, these results argue that the ultimate theoretical potential of HAART to control viral replication has already been reached. If the therapeutic goal is virus eradication, then novel strategies need to be adopted to target and clear the latent reservoirs. This clearance could be achieved by inducing HIV-1 replication in latently-infected cells, while maintaining or intensifying HAART in order to prevent new spreading infection. Once reactivated, latently-infected cells will be eliminated by the host immune system and/or virus-mediated cell lyses. It should be kept in mind that a single residual latently-infected cell can in theory rapidly rekindle the infection. However, a decline of the HIV-1 reservoir to a level sufficient to allow an efficient control of the infection by the host immune system might allow for interruptions in therapy ("treatment-free windows") and would represent important progress in the treatment of HIV-1.

This review focuses on our current knowledge and understanding of the molecular mechanisms involved in HIV-1 transcriptional latency, whose deeper comprehension could lead to new therapeutic strategies aimed towards combining HIV-1 gene expression activators with an effective HAART for decreasing/eradicating the pool of latently-infected cells. We will detail the more advanced treatment strategies based on T-cell activation and HDAC inhibitors, and also discuss the still-in-progress concepts such as potential treatments targeting Tat-associated factors and DNA- and histone- methylation.

## Pre- and postintegration latency

Two general forms of viral latency have been observed and can be segregated based on whether or not the virus has integrated into the host cell genome: preintegration and postintegration latency (reviewed in [36,37]). Preintegration latency results from partial or complete block of the viral life cycle at steps prior to the integration of the virus into the host genome [30,38]. This block could result from incomplete reverse transcription as a result of a reduced dNTP pool in metabolically inactive cells [39] or from restriction by factors such as APOBEC3G, a cellular deoxycytidine deaminase whose action can be counteracted by the viral Vif protein [40-43]. The preintegration complex (PIC) could also fail to be imported into the nucleus owing to a lack of ATP [44]. Among cellular restriction factors of retroviral replication, TRIM5α trimers from Old World Monkeys but not from humans restrict HIV-1 infection, probably by disrupting the uncoating of virion cores and interrupting the subsequent intracellular trafficking needed for proviral DNA to enter the nucleus [45-47]. While linear unintegrated DNA is suceptible to integration into the host cell genome following activation [44], preintegration latency does not appear to be of clinical relevance because of its labile nature in T cells (unintegrated forms persist in the cytoplasm of these cells for only one day and cannot account for the formation of long-term latently-infected CD4^+ ^T-cell reservoirs) [48-50]. Of note, unintegrated DNA remains stable for at least one month in non-dividing but metabolically active macrophages [51,52], and seems to maintain biological activity [53]. Most studies on preintegrated (and postintegrated) forms of HIV-1 have been conducted in proliferating T cells. In order to be clinically relevant, these studies should be extended to other natural host cells of the virus (such as macrophages and microglial cells).

**Figure 1:**
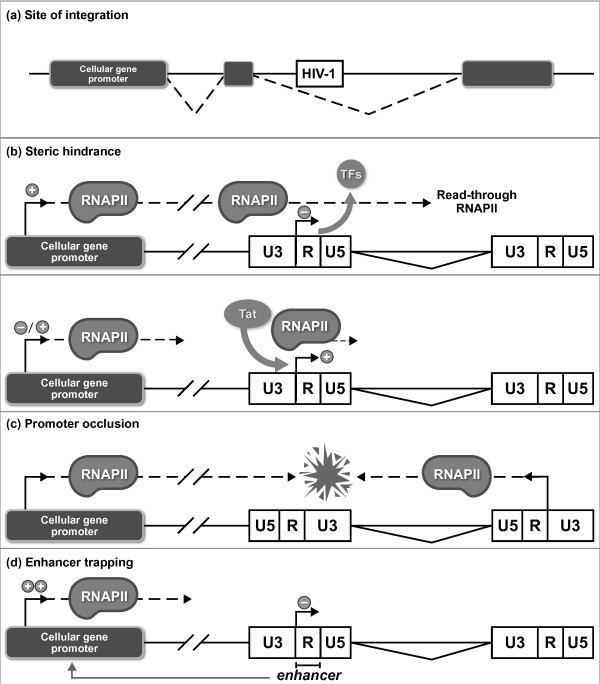
**A simplified view of the multiple mechanisms of transcriptional interference implicated in HIV-1 postintegration latency**. (a) HIV-1 integrates into the host cell genome predominantly in intronic regions of actively transcribed genes [55-57]. Transcriptional interference may lead to the establishment of latency by different mechanisms depending at least on the orientation of viral integration compared to the host gene. (b) *steric hindrance*: when proviral integration occurs in the same transcriptional orientation as the cellular host gene, "read-through" RNA polymerase II (RNAPII) transcription from the upstream promoter displaces key transcription factors (TFs) from the HIV-1 promoter [60] and prevents assembly of the pre-initiation complex on the viral promoter. The integrated virus is thought to be transcribed along with the other intronic regions of the cellular gene, but is then merely spliced out. HIV-1 transcription inhibition could be reversed by hindering the upstream transcription or by cooperatively activating viral transcription initiation and elongation; certain host transcription factors and/or viral activators, which bind strongly to their cognate site, could resist the "read-through" RNAPII passage [61]. This phenomenon was also observed following Tat-mediated transactivation of HIV-1 transcription [67]. (c) *promoter occlusion*: provirus integration in the opposite orientation compared to the host gene may lead to collisions of the elongating RNA polymerases from each promoter, resulting in a premature termination of transcription from the weaker or from both promoters. (d) *enhancer trapping*: an enhancer of one gene (the 5'LTR *enhancer *of HIV-1 in this case) is placed out of context near the promoter of a second gene (a cellular gene in this case) and acts on the transcriptional activity of this cellular promoter, thereby preventing the enhancer action on the 5'LTR.

Postintegration latency occurs when a provirus fails to effectively express its genome and is reversibly silenced after integration into the host cell genome. This latent state is exceptionally stable and is limited only by the lifespan of the infected cell and its progeny. Several aspects contribute to the transcriptional silencing of integrated HIV-1 proviruses:

*(1) The site of integration*. HIV-1 integrates into the host chromosomal DNA in a non-random manner. Following nuclear import, LEDGF/p75, a transcriptional coactivator which interacts directly with the viral integrase [54], targets the PIC predominantly to intronic regions of actively transcribed genes [55-57]. An analysis of integration sites in purified resting CD4^+ ^T cells from infected patients on HAART found that the majority (93%) of silent proviruses is located within the coding region of host genes [58], although it is unclear whether these integration events are representative of defective proviruses or reflect true latency [13]. The finding that latent HIV-1 proviruses integrate in actively transcribed regions may seem paradoxical considering the establishment of a transcriptionally latent state. However, several different mechanisms of transcriptional interference may clarify this point (reviewed in [36] and [59]) (see Fig 1): (i) Steric hindrance: when the provirus integrates in the same transcriptional orientation as the host gene, "read-through" transcription from an upstream promoter displaces key transcription factors from the HIV-1 promoter as previously shown for Sp1 [60] and prevents the assembly of the pre-initiation complex on the viral promoter, thereby hindering HIV-1 transcription. The integrated virus is thought to be transcribed along with the other intronic regions of the cellular gene, but is then merely spliced out. This mechanism has been confirmed in J-Lat cells, a CD4^+ ^T-cell line used as a model for HIV-1 postintegration latency [61]. Lenasi and colleagues have shown that transcriptional interference could be reversed by inhibiting the upstream transcription or by cooperatively activating viral transcription initiation and elongation. Of note, certain host transcription factors and/or viral activators, which bind strongly to their cognate sites, could resist the passage of "read-through" RNA polymerase II (RNAPII) [61]. As studies in yeast have demonstrated that the elongating polymerase is followed by a rapid refolding of histones in a closed configuration to counteract transcription initiation at cryptic sites in the transcription unit [62], chromatin structure and epigenetic events could also be implicated in transcriptional interference. Conversely, Han *et al*. [63] have demonstrated that upstream transcription could enhance HIV-1 gene expression without significant modification of the chromatin status in the region when the provirus is integrated in the same orientation as the host gene. These partially contradictory studies have been questioned [64] based on earlier studies that reported transcriptional interference as important in repressing viral promoters integrated in the same orientation as an upstream host gene promoter [60,65,66]. Interestingly, Marcello and colleagues [67] have recently reported that an integrated provirus suffering from transcriptional interference in basal conditions becomes transcriptionally active following Tat expression; and that this provirus can switch off the transcription of the host gene within which it has integrated or can allow the coexistence of expression of both host and viral genes. Further analysis of the mechanisms exploited by host genes to regulate a viral promoter inserted in their transcriptional unit or by the virus to counterbalance the host gene control will be needed to completely elucidate these transcriptional interference events. (ii) Promoter occlusion: provirus integration in the opposite orientation to the host gene may lead to the collision of elongating polymerases from each promoter, resulting in a premature termination of transcription from either the weaker or both promoters [66,68]. Convergent transcription may also allow for the elongation of both viral DNA strands. The subsequent formation of double-stranded RNAs might lead to RNA interference, RNA-directed DNA methylation or generation of antisense RNAs [69]. (iii) Enhancer trapping: this phenomenon can occur when an enhancer of one gene is placed out of context near the promoter of a second gene.

The spatial distribution of genes within the nucleus contributes to transcriptional control, allowing for constitutive or regulated gene expression. In this regard, a recent study has demonstrated a correlation between HIV-1 provirus transcriptional repression and its interaction with a pericentromeric region of chromosome 12 in several clones of J-Lat cells [70]. In general, heterochromatin lines the inner surface of the nuclear envelope, whereas transcriptionally active euchromatin is dispersed in the nuclear core. Here, however, the peripheral localization of the provirus was observed even after induction, suggesting that certain portions of the nuclear periphery could provide an environment allowing reversible silencing [70].

*(2) The pool of available cellular transcription factors*. The 5'LTR functions as the HIV-1 promoter and contains binding sites for several ubiquitously expressed transcription factors, such as Sp1 and TFIID, and inducible transcription factors, including NF-κB, NFAT and AP-1. HIV-1 transcription is tightly coupled to the cellular activation status because both NF-κB and NFAT are sequestered in the cytoplasm of quiescent T cells and recruited to the nucleus following T-cell activation. The relevance of these (and other) transcription factors in a potential therapeutic strategy based on reactivation of HIV-1 latently-infected cells is discussed below.

*(3) The chromatin organization of the HIV-1 promoter*. Two nucleosomes, namely nuc-0 and nuc-1, are precisely positioned in the promoter region of HIV-1 in latently-infected cell lines [71,72] and impose a block to transcriptional elongation. Following transcriptional activation, nuc-1 (located immediately downstream of the transcription start site) is specifically remodeled [73]. The mechanisms underlying maintenance of a repressive chromatin state of the HIV-1 provirus in latently-infected cells and the factors implicated in the remodeling of nuc-1 will be further discussed in association with epigenetic modifications of the HIV-1 5'LTR region (posttranslational modifications of the histone N-terminal tails in the promoter region and DNA methylation status).

*(4) The viral protein Tat and Tat-associated factors*. In addition to the need for host transcription factors binding to their cognate sites in the 5'LTR, HIV-1 transcription is boosted by the viral trans-activating protein Tat, which interacts with the *cis*-acting RNA element TAR (Transactivation response element) present at the 5'end of all nascent viral transcripts. Several host factors, including Cdk9, Cyclin T1 and histone acetyltransferases, are then recruited by Tat to unravel the transcriptional block at the early elongation stage. Tat itself or Tat-associated proteins could be limiting factors for processive transcription in resting T cells, thereby inducing a latent HIV-1 infection. These limiting factors are further discussed below.

*(5) MicroRNAs and RNA interference*. MicroRNAs (miRNAs) are single-stranded noncoding RNAs of 19 to 25 nucleotides in length that function as gene regulators and as a host cell defense against both RNA and DNA viruses [74]. Primary miRNAs are sequentially processed via the nuclear RNases III Drosha and Dicer to generate mature miRNAs which interact with a complementary sequence in the 3' untranslated region of target mRNAs by partial sequence matching, resulting in degradation of the mRNA and/or translational inhibition [75]. Recent publications demonstrate that miRNAs can also regulate gene expression at the epigenetic level, by specifically inducing methylation along the promoter region or by directly generating the remodeling of the surrounding chromatin [76,77]. The RNA interference pathway constitutes an additional level of complexity to the viral-host interplay. First, a cluster of cellular miRNAs was found to be enriched specifically in resting CD4^+ ^T cells using microarray technology and has been shown to suppress translation of most HIV-1-encoded proteins (including Tat and Rev, but not Nef), thereby sustaining HIV-1 escape from the host immune response [78]. More recently, the cellular miRNA hsa-miR29a has been demonstrated to downregulate the expression of the Nef protein and, in that way, to interfere with HIV-1 replication [79]. Moreover, several cellular factors required for miRNA-mediated mRNA translational inhibition have been characterized as negative regulators of HIV-1 gene expression [80]. Second, HIV-1 can suppress the miRNA-mediated silencing pathway during infection of cells. Thus, by reducing the expression of some cellular miRNAs (e.g. miR-17-5p and 20a) the virus can increase the expression of the Tat cofactor PCAF (which is otherwise normally silenced by the miR-17-5p miRNA cluster) and promote viral transcription [81]. Alternatively, HIV-1 transcripts (such as TAR and nef) can be processed into miRNAs (nef [82,83] and TAR [84,85]), which have been suggested to contribute in part to establishing a latent state by directly downregulating HIV-1 transcription or by indirectly recruiting HDACs to the 5'LTR promoter. There are also reports that HIV-1 infection can modulate cellular RNA-interference (RNAi) activity through the viral Tat protein [86,87] and the TAR RNA [88], notably by moderating DICER activity. The usefulness of RNAi as a potential intervention against HIV-1 replication has been provocatively suggested by Suzuki *et al*. [89] who have employed siRNA targeted against NF-κB-sequences in the HIV-1 LTR to enforce transcriptional gene silencing (TGS). Indeed, there is a complex interplay between HIV-1 replication and the cell's RNAi pathways. The potential utility of this virus-host interaction relevant to eradicating latent viral reservoirs has been reviewed elsewhere ([90] and [91]).

## In vitro models for HIV-1 postintegration latency

Postintegration latency is established within days following acute infection when productively-infected CD4^+ ^T cells revert to the resting state, becoming memory T cells. As discussed above, the molecular mechanisms involved in the establishment and maintenance of latency are multifactorial and involve many elements of HIV-1 transcription. Unfortunately, the study of latency *in vivo *has been hampered by the scarcity of latently-infected cells (0.1-1 infected cell per million CD4^+ ^lymphocytes [13]), their difficult enrichment due to the lack of any viral marker (avoiding antibody-based purification strategies), and the high background rate of defective integrated proviruses. Cell culture model systems have been generated (including the ACH2 T-cell line [92] and the promonocytic U1 cell line [93,94]) which show minimal constitutive expression of HIV-1 genes, but a marked activation of viral gene expression following treatment with cytokines or mitogens. These models have revealed many early insights into the mechanisms of HIV-1 latency, despite the fact that mutations in Tat (U1) [95] or in its RNA target TAR (ACH2) [96] have been demonstrated to be causative of the latent phenotype of the proviruses integrated in these two cell lines. More recently, J-Lat cells were developed with an HIV-1-based vector containing an intact Tat/TAR axis [97]. These cells whose unique provirus carries the coding sequence for green fluorescent protein (GFP) instead of the *nef *gene were selected for a lack of GFP expression under basal conditions [97]; they allow for the rapid assessment of HIV transcriptional activity by cytometric detection of GFP epifluorescence. As an alternative, Ben Berkhout's laboratory has developed stable cell lines containing an HIV-rtTA variant (in which the Tat/TAR axis transcription motifs have been inactivated and replaced by the inducible Tet-ON system [98]). The HIV-rtTA provirus is completely doxycycline-dependent for virus production; it contains the original transcription factor binding sites in the HIV 5'LTR, and infected cells have been obtained without selection steps avoiding any bias towards activation markers [99]. However, the constantly activated and proliferating nature of infected cell lines does not accurately represent the quiescent cellular environment of latently-infected cells *in vivo *and the improvement of new models nearer to the *in vivo *situation is an important goal for HIV-1 research [100]. Interestingly, new *ex vivo *experimental systems based on primary human CD4^+ ^T cells or primary derived macrophages were recently developed to study HIV-1 latency in a more physiological context [101-104]. Among those, Bosque and Planelles infected memory CD4^+ ^T cells (obtained from naïve T cells purified from healthy donors and activated under conditions that drive them to become memory T cells) with a virus defective in Env, which was then provided in *trans *[103]. Of note, these cells were kept in culture in the presence of IL-2, what could disturb the quiescent state of the cells. Separately, Siliciano's group developed a new *in vitro *model of HIV-1 latency using human primary CD4^+ ^T cells [104]. These cells were transduced with the anti-apoptotic protein Bcl-2 to ensure the survival of memory CD4^+ ^T cells and infected with a modified HIV-1 vector in order to increase the yield of latently-infected cells. The modified HIV-1 vector preserves LTR, *tat *and *rev *genes, and the signaling pathways leading to viral reactivation are intact. Thus, this model can be used to study the reactivation of HIV-1 from latency. Collectively, these new models may be helpful to address the mechanisms implicated in the switch from productive to latent infection and *vice versa*, even if they remain technically difficult to establish and maintain.

## T-cell activation-mediated transcription factors involved in HIV-1 transcription

**Figure 2:**
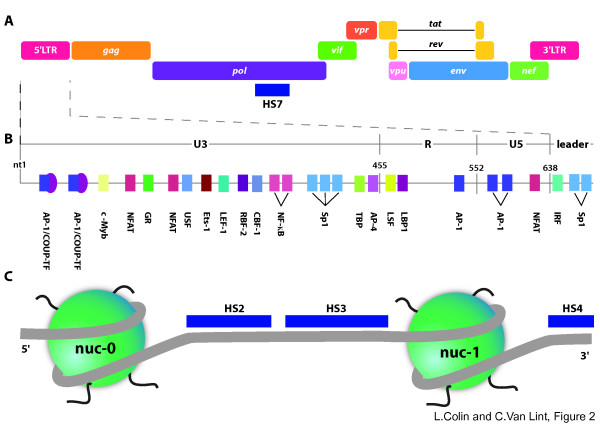
**Transcription factor binding sites and chromatin organization in the 5'LTR and leader region of HIV-1**. (A) Representation of the HIV-1 genome. The intragenic hypersensitive site HS7 located in the *pol *gene is indicated. (B) Schematic representation of the main transcription factor binding sites located in the 5'LTR and in the beginning of the leader region of HIV-1. The U3, R, U5 and leader regions are indicated. Nucleotide 1 (nt1) is the start of U3 in the 5'LTR. The transcription start site corresponds to the junction of U3 and R. (C) Schematic representation of the nucleosomal organization of the HIV-1 genome 5' region. Hypersensitive sites HS2, HS3 and HS4 are indicated. The assignment of nucleosome position in this region is based on DNase I, micrococcal nuclease and restriction enzyme digestion profiles [72,73]. During transcriptional activation, a single nucleosome, named nuc-1 and located immediately downstream of the transcription start site, is specifically and rapidly remodeled [73].

The 5'LTR of HIV-1 contains several DNA-binding sites for various cellular transcription factors, including Sp1 and NF-κB binding sites which are required for HIV-1 replication [105,106], whereas other sites, such as NFAT, LEF-1, COUP-TF, Ets1, USF and AP-1 binding sites, enhance transcription without being indispensable (see Fig 2B).

**Figure 3:**
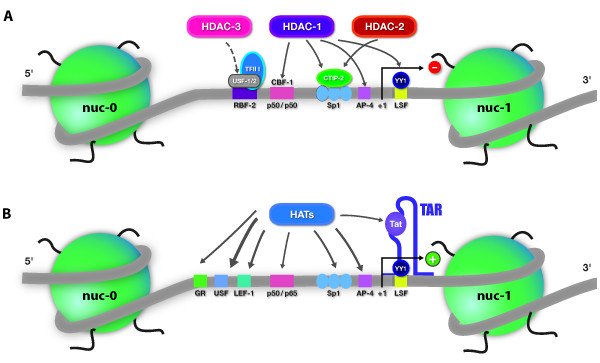
**HDAC and HAT recruitment to the HIV-1 5'LTR**. (A) During latency, nuc-1 blocks transcriptional initiation and/or elongation because it is maintained hypoacetylated by nearby recruited HDACs. The targeting of nuc-1 by these HDACs is mediated by their recruitment to the 5'LTR via several transcription factor binding sites. Thin arrows indicate that the implicated transcription factors were demonstrated to recruit HDACs to the 5'LTR (by ChIP experiments or following knock-down of the corresponding transcription factor). The dotted arrow indicates that the USF transcription factor could potentially recruit HDAC-3 to the nuc-1 region based on its interactome partners in the literature, but this recruitment has not been demonstrated so far in the specific context of the HIV-1 promoter. (B) Nuc-1 is a major obstacle to transcription and has to be remodeled to activate transcription. This disruption could happen following local recruitment of HATs by DNA-binding factors, and/or by the viral protein Tat, which binds to the neo-synthesized TAR element. This would result in nuc-1 hyperacetylation and remodeling, thereby eliminating the block to transcription at least for certain forms of viral latency. This acetylation-based activation model has been validated notably regarding the involvement of the transcription factors NF-κB p65 and Tat.

NF-κB, typically a p50/p65 heterodimer, is sequestered in the cytoplasm of unstimulated cells in an inactive form through its interaction with an inhibitory protein from the family of inhibitors of NF-κB (IKB). Following activation of the protein kinase C (PKC) pathway, phosphorylation of IKB by IKK (IKB kinase) leads to its dissociation from NF-κB and its subsequent polyubiquitination and degradation by the proteasome pathway. This dissociation allows NF-κB translocation into the nucleus, and the transcriptional trans-activation of NF-κB-dependent genes. In resting CD4^+ ^T cells, both IκBα and NF-κB are continuously shuttling between the cytosol and the nucleus, as well as continuously associating and dissociating; these fluctuations can impact HIV-1 transcription in these cells [107]. In HIV-1 latently-infected cells, NF-κB p50/p50 homodimers, which lack the trans- activation domain found in the p50/p65 heterodimer, recruit the histone deacetylase HDAC-1 to the LTR, leading to local histone deacetylation and to a repressive chromatin structure in the HIV-1 5'LTR [108] (Fig 3A). Following T-cell activation, p50/p50 homodimers are displaced by liberated cytoplasmic stores of p50/p65 heterodimers, which in turn recruit histone acetyltransferases (HATs) (such as CBP and p300), thereby driving local histone acetylation [109-112] to enhance transcription (Fig 3B). NF-κB activity itself is modulated by direct posttranslational acetylation of the p65 and p50 subunits. These modifications affect several NF-κB functions, including transcriptional activation, DNA-binding affinity and IKBα assembly [113,114]. The p65 subunit of NF-κB additionally stimulates transcriptional elongation by interacting with RNAPII complexes including Cdk7/TFIIH [115] and pTEFb [116]. TFIIH/Cdk7 and pTEFb direct the phosphorylation of serine-5 and serine-2 residues, respectively, in the carboxy-terminal domain (CTD) of the RNAPII. These phosphorylation events are necessary to allow promoter clearance and efficient transcriptional elongation by RNAPII. Interestingly, a siRNA targeting conserved tandem NF-κB motifs in the HIV-1 5'LTR was associated with increased CpG methylation in the 5'LTR and was shown to suppress viral replication in chronically infected MAGIC-5 cells [89]. The recruitment of transcriptional silencing machinery via this siRNA targeted to NF-κB binding site sequences seems to correlate with transcriptional silencing and HIV-1 latency [89].

In response to TCR-triggered Ca^2+ ^release via the PKC pathway, cytoplasmic NFAT is rapidly dephosphorylated by calcineurin and translocates into the nucleus [117]. NFAT interacts with the 5'LTR at sites overlapping the U3 NF-κB binding sites, suggesting mutually exclusive binding and alternate transactivation by these two factors [118]. A NFAT downstream binding site was also characterized in the U5 region of the viral 5'LTR [119,120] (Fig 2B). Recruitment of the coactivators p300 and CBP by the transactivation domains of NFAT proteins [121] suggests that, like NF-κB, members of the NFAT family could promote chromatin remodeling of the HIV-1 5'LTR. T-cell receptor pathway also induces AP-1 dimers, composed of members of the Jun, Fos and ATF families, by activation of c-Jun N-terminal kinase (JNK) and extracellular signal-related kinase (ERK) [122,123]. Studies of host NFAT-responsive promoters indicate that NFAT binding induces extensive nucleosomal disruption, in a manner dependent on cooperative binding with AP-1 [124]. Moreover, Tat interacts with NFAT, increasing its cooperation with AP-1, without altering independent binding of the AP-1 transcription factors to DNA [125]. These results suggest that AP-1 can cooperate with NFAT to activate HIV-1 transcription through the U3 NF-κB/NFAT binding sites.

Our laboratory has also identified binding sites for NFAT, AP-1 and other transcription factors downstream of the transcription start site (Fig 2B) [120], in a large nucleosome-free region where we had previously identified a DNase-I hypersensitive site named HS4 [71,120] (Fig 2C). These downstream binding sites include three AP-1 binding sites, a NFAT motif, an interferon-responsive factor (IRF) binding site, and two juxtaposed Sp1 sites, which are important for viral infectivity [120]. The NFAT motif lies at the 3' boundary of the nucleosome nuc-1 and may play a role in nuc-1 remodeling observed following T-cell activation [126]. The HS4 binding sites constitute an enhancer that could function independently of, or in concert with, other factors binding to the HIV-1 5'LTR in order to activate HIV-1 transcription [120].

Analysis of the chromatin organization of integrated HIV-1 proviruses identified a major hypersensitive site in the region of 8 kb between the two LTRs. This hypersensitive site, named HS7 and encompassing nt 4481-4982 (where nt+1 is the transcription start site) (Fig 2A), is located in the *pol *gene between two subdomains (termed the 5103 and the 5105 fragments), both exhibiting phorbol ester-inducible enhancing activity in HeLa cells [71]. The HS7 site is present only in the U1 cell line of monocyte/macrophage origin, and not in the ACH2 and 8E5 cell lines of T-cell origin. A 500 bp fragment including HS7 positively regulates transcription from the 5'LTR in transient transfection experiments conducted using T- or monocytic- cell lines [127]. Multiple transcription factor binding sites have been identified in the HS7 region. These include ubiquitously expressed transcription factors such as Sp1/Sp3, Oct1 and AP-1 and cell-specific transcription factors such as PU.1, which is only expressed in the monocyte/macrophage and B-cell lineages [128]. Three AP-1 binding sites have also been characterized in the 5103 fragment [129], and our laboratory has recently shown that these sites are important for viral infectivity (unpublished results). An additional AP-1 binding site and an Ets-1 binding site were identified in the 5105 fragment (unpublished data from our laboratory). Interestingly, Ets-1 was recently shown to reactivate latent HIV-1 in an NF-κB independent manner in a strategy based on transcription factor expression in order to avoid general T-cell activation [130]. The intragenic regulatory region (whose complete functional unit is composed of the 5103 fragment, the HS7, and the 5105 fragment) represents an additional factor in an already complex network of regulation that affects HIV-1 transcription.

## PKC agonists to induce HIV-1 latent reservoirs

Signaling through PKC was considered as an interesting pathway to induce latent proviral expression because of the multiplicity of transcription factor binding sites for NF-κB, NFAT and AP-1 in the HIV-1 5'LTR and the *pol *gene intragenic region. New PKC agonists, including synthetic analogs of diacylglycerol [131], ingenols [132], phorbol-13-monoesters [133], a jatrophane diterpene (named SJ23B) [134], and the two non tumorigenic phorbol esters prostratin [135,136] and DPP (12-deoxyphorbol 13-phenylacetate) [137], have proven capable of inducing HIV-1 transcription in latently-infected CD4^+ ^T cells or in PBMCs (peripheral blood mononuclear cells) from HAART-treated patients. PKC agonists down-regulate the expression of the HIV-1 receptor CD4 and the coreceptors CXCR4 and CCR5 on the host cell surface [132,138,139]. Therefore, these compounds exhibit interesting bipolar properties as potential molecules to purge resting T-cell latent reservoirs: they upregulate the expression of latent proviruses and inhibit the spread of newly synthesized viruses to uninfected cells via down-regulation of critical receptors necessary for viral entry [140].

The phorbol ester prostratin, found to be the active agent used by Samoan tribesmen to treat jaundice, is extracted from the plant *Homolanthus nutans *[141]. It activates HIV-1 expression in latently-infected lymphoid and myeloid cell lines and in primary cells [135-137,139-142] with minimal effects on the immune system [141] and causes minimal perturbation of cell cycle progression [142]. Like bryostatin 1 and DPP, prostratin is an interesting compound as a PKC activator without tumor-promoting activity. The non-mitogenic property of prostratin, its remarkable dual role in activating HIV-1 latently-infected reservoirs without spreading infection, its relatively non- toxic behavior, and its ability to act on different cell types make this drug a good candidate for viral purging. Despite these numerous advantages, the use of prostratin (and DPP) in human clinical trials awaits safety and toxicity studies in a suitable primate model [143,144]. However, preliminary pharmacokinetic studies are encouraging [135]. Furthermore, chemical synthesis of this therapeutically promising natural compound in gram quantities and at low cost was recently reported [145]; this efficient method of synthesis promises to open the access to numerous new analogs.

In conclusion, strategies to purge viral reservoirs with PKC agonists are dependent, at least in part, on the induction of the cellular transcription factors NF-κB and NFAT/AP-1 by the PKC pathway. These transcription factors bind to their cognate binding sites in the 5'LTR and in the intragenic region of HIV-1 to activate transcription of latent proviruses.

## T-cell activation as a strategy against HIV-1 latency: Immune Activation Therapy

There has been considerable interest in the possibility that eradication of latent reservoirs might be feasible through global cellular activation [146-148]. This strategy is termed immune activation therapy (IAT). The achievability of cytokine-based IAT was proven *in vitro *with a combination of the pro-inflammatory cytokines interleukin-6 (IL-6) and TNF-α, along with the immunoregulatory cytokine interleukin-2 (IL-2), a combination which was a potent inducer of viral replication in latently-infected CD4^+ ^resting T cells isolated from therapy-naïve as well as HAART-treated patients [149]. Several studies with patients cotreated with HAART and IL-2 administration have shown a reduction of CD4^+ ^T cells containing replication-competent HIV-1 proviruses [150-152]. However, in these studies, the reemergence of plasma viremia and of the latent pool within the 2-3 weeks following treatment interruption suggested that only a partial purge of latent reservoirs had been reached [150-152]. To additionally affect HIV-1-infected monocyte/macrophage cells, gamma-interferon (IFN- γ) was added to IL-2, but a similar rebound of viremia was observed after ceasing treatment [153]. Later studies attempted to improve the results of therapy using IL-2 and HAART with the OKT3 antibody, which binds the T-cell receptor complex, in order to deplete T cells [154]. Upregulation of HIV-1 expression occurred but no demonstrable effect toward purging latent reservoirs could be obtained [155,156]. In these latter studies, treated patients experienced over the long term considerable CD4^+ ^T cell depletion, which was not reversible after treatment interruption [157], and might compromise immunity. These patients additionally developed severe side effects linked to the appearance of anti-OKT3 antibodies due to its murine origin. The side effects were avoided by the administration of lower doses of OKT3, leading to a clinically more successful study where the spectrum of viral genotypes among the rebounding viruses differed significantly from isolates recovered at the beginning of the study [147]. This modulation of the viral pool suggested that the activation of latent proviruses had happened, but a rebound of plasma viremia still occurred several weeks after therapy [147].

Using latently-infected cells generated in the SCID/hu mice model, Brooks *et al*. have reported that IL-7 is able to reactivate latent HIV-1 viruses [142]. Moreover, IL-7 has been shown to induce the *in vitro *expression of latent HIV-1 proviruses in resting CD4^+ ^T cells from HIV-infected patients under HAART treatment [158,159]; and its therapeutic potential has been attested based on biologic and cytotoxicity profiles [160,161]. However, IL-7, such as other cytokines, induces the proliferation and survival of CD4^+ ^memory T cells [162], and this property enables a quantitatively stable pool of latently-infected memory CD4^+ ^T cells to be maintained in HAART-treated individuals [163,164]. Importantly, Chomont *et al*. [163] have very recently shown that different mechanisms ensure viral persistence in the central memory T cells (T_CM_) compared to transitional memory T cells(T_TM_). In the first cell population, the HIV-1 reservoir persists through cell survival and low-level antigen driven proliferation. This situation is observed in HAART-treated patients with high CD4^+ ^levels. In the second cell population, mainly representative of the situation in aviremic patients with low CD4^+ ^levels, homeostatic proliferation and subsequent persistence of the cells mediated by IL-7 is implicated in the maintenance of latent reservoirs. These results incriminate IL-7 specifically (and cytokines in general) in the maintenance of a reservoir of latently-infected CD4^+ ^T cells [163], thereby questioning the relevance of immune activation therapy in the context of a purge of latently-infected reservoirs in HAART-treated patients.

## Chromatin structure and epigenetic regulation of eucaryotic gene expression

In eukaryotic cells, DNA is packaged within chromatin to allow the efficient storage of genetic information. The structural and functional repeating unit of chromatin is the nucleosome, in which 146 DNA base pairs are tightly wrapped in 1.65 superhelical turns around an octamer composed of two molecules of each of the four core histones H2A, H2B, H3 and H4 [165]. Each nucleosome is linked to the next by small segments of linker DNA, and the polynucleosome fiber might be stabilized by the binding of histone H1 to each nucleosome and successive DNA linker. Chromatin condensation is critical for the regulation of gene expression since it determines the accessibility of DNA to regulatory transcription factors. Euchromatin corresponds to decondensed genome regions generally associated with actively transcribed genes. By contrast, heterochromatin refers to highly condensed and transcriptionally inactive regions of the genome [166].

The chromatin condensation status can be modulated through a variety of mechanisms, including posttranslational covalent modifications of histone tails and ATP-dependent chromatin remodeling events [167,168]. ATP-dependent chromatin remodeling complexes couple the hydrolysis of ATP to structural changes of the nucleosome and are divided into three main classes based on their ATPase subunit: the SWI/SNF family, the ISWI family and the Mi-2 family [169]. Histone modifications are all reversible and mainly localize to the amino- and carboxy-terminal histone tails. They include acetylation, methylation, phosphorylation, sumoylation, ADP-ribosylation and ubiquitination. These covalent modifications of histone tails influence gene expression patterns by two different mechanisms [170]: (1) by directly altering chromatin packaging, electrostatic charge modifications or internucleosomal contacts might emphasize or reduce the access of DNA to transcription factors; (2) by generating interactions with chromatin-associated proteins. These modifications function sequentially or act in combination to form the "histone code" and serve as extremely selective recruitment platforms for specific regulatory proteins that drive different biological processes [171].

Histone acetyltransferases (HATs) and histone deacetylases (HDACs) influence transcription by selectively acetylating or deacetylating the ε-amino groups of lysine residues in histone tails. Generally, chromatin acetylation by HATs promotes chromatin opening and is associated with active euchromatin, whereas deacetylation by HDACs diminishes the accessibility of the nucleosomal DNA to transcription factors, thereby generating repressive heterochromatin [172]. Moreover, histone acetylation marks enable the recruitment of bromodomain-containing proteins, such as chromatin remodeling complexes and transcriptions factors, which in turn regulate gene expression. HATs and HDACs are usually embedded in large multimolecular complexes, in which the other subunits function as cofactors for the enzyme [173]. They are also involved in the reversible acetylation of non-histone proteins [174]. Humans HDACs have first been classified into three classes, based on their homolog in yeast (see table 1, panel a): class I (HDACs 1, 2, 3 and 8), class II (subdivided into class IIa: HDACs 4, 5, 7, 9 and class IIb: HDAC 6, 10), and class III (Sirt1 - Sirt7) are homologs of yeast RPD3, Hda1 and Sir2, respectively [175]. HDAC-11 is most closely related to class I, but was classified alone into class IV because of its low sequence similarity with the other members of class I HDACs. HATs have also been grouped into different classes based on sequence homologies and biological functions (see table 1, panel b): the Gcn5-related N-acetyltransferases (GNATs), the p300/CBP and the MYST protein families, while several other not yet classified proteins (such as transcription factors and nuclear receptor coactivators) have been reported to possess HAT activity [176].

**Table 1:** Chromatin-modifying enzymes.

a. HDACs (Histone deacetylases)	
**Family**	**Members**

Class I	**HDAC-1**; **HDAC-2**; **HDAC-3**; HDAC-8

Class Iia	HDAC-4; HDAC-5; HDAC-7; HDAC-9

Class IIb	HDAC-6; HDAC-10

Class III	**SIRT1**; SIRT2; SIRT3; SIRT4; SIRT5; SIRT6; SIRT7

Class IV	HDAC-11

**b. HATs (Histone acetyltransferases)**	

**Family**	**Members**

GNAT	**Gcn5**; ELP3; HAT1; **PCAF**

p300/CBP	**p300**; **CBP**

MYST	ESA1; CLOCK; MOF/MYST1; HBO1/MYST2; MOZ/MYST3/HAT3; MORF/MYST4; SAS2; TIP60; YBF2/SAS3

Others	ACTR; ATF-2; GRIP; p/CIP; SRC1; TAF1; TFIIB

**c. HMTs (Histone methyltransferases)**	

**Family**	**Members**

HKMTs	ALL-1; ALR; AsH1; DOT1L; **ESET/SETDB1**; EuHMTase/GLP; EZH2; **G9a**; MLL1; MLL2; MLL3; MLL4; MLL5; NSD1; RIZ1; SET1; SET2; SET 7/8; SET7/9; SMYD2; SMYD3; **Suv39 h1**; Suv39H2; Suv4-20H1; Suv4-20H2

PRMTs	PRMT1; PRMT2; PRMT3; PRMT4/CARM1; PRMT5/JBP1; **PRMT6**

**d. HDMTs (Histone demethylases)**	

**Family**	**Members**

LSD1	**LSD1**

JHDM/Jumonji	JARID1A/RBP-2; JARID1B/PLU-1; JARID1C/SMCX; JARID1D/SMCY; JHDM1a; JHDM1b; JHDM2a; JHDM2b; JMJD2A; JMJD2B; JMJD2C; JMJD2D; JMJD3

**e. DNMTs (DNA methyltransferases)**	

**Family**	**Members**

Maintenance	**DNMT1**

	DNMT2

*De novo*	**DNMT3a**; DNMT3b; DNMT3l

This table summarizes the main family members of HDACs (panel a), HATs (panel b), HMTs (panel c), HDMTs (panel d) and DNMTs (panel e). To facilitate comprehension, enzymes that appear in the text of this review are indicated in bold.

Abbreviations used in table 1: ACTR: Activator of thyroid and retinoic acid receptors; ALL-1: acute lymphoblastic leukemia; ALR: ALL-*1 *related gene; AsH1: absent small or homeotic discs1; ATF-2: Activating Transcription Factor; CARM1: coactivator-associated arginine methyltransferase 1; CBP: CREB binding protein; DOT1: disrupter of telomeric silencing; ELP3: elongation protein 3 homolog; ESET: SET domain bifurcated 1; Eu-HMTase: euchromatic histone methyltransferase 1; EZH2: enhancer of zeste homolog 2; Gcn5: General control nuclear factor 5; GNAT: Gcn5-related N-AcetylTransferase; GRIP: glucocorticoid receptor-interacting protein 1; HBO1: HAT bound to DNA replication origin complex; HKMT: Histone methyltransferase; JARID: Jumonji, AT rich interactive domain; JHDM: JmjC domain containing histone demethylases; JMJD: Jumonji-domain containing; LSD1: lysine-specific demethylase 1; MLL: Mixed-lineage leukemia; JBP1: Janus kinase binding protein 1; MOF: males absent on the first; MORF: MOZ-related factor; MOZ: Monocyte leukemia zinc-finger protein; MYST: named for its founding members MOZ, *YBF2*/SAS3, SAS2, and Tip60; NSD1: nuclear receptor binding SET domain protein 1; p/CAF: p300/CBP associated factor; p/CIP: p300/CBP interacting protein; PRMT: Protein arginine methyltransferase; RBP-2: retinol-binding protein 2; RIZ1: retinoblastoma protein-interacting zinc-finger 1; SAS2: serum antigenic substance 2; SET1: SET domain containing 1; SETDB1: SET domain bifurcated 1; SIRT: Sirtuin; SMC: Structural Maintenance of Chromosomes-1; SMYD: SET- and MYND-domain-containing; SRC: Steroid receptor coactivator; Suv39 h1: suppressor of variegation 3-9 homolog 1; Suv4-20 h1: suppressor of variegation 4-20 h1; TAF1: TATA-binding protein (TBP)-associated factor; TFIIB: Transcription factor IIB; TIP60: Tat interacting protein (60 kD).

Histone lysine methyltransferases (HKMTs) and protein arginine methyltransferases (PRMTs) catalyze the transfer of one to three methyl groups from the cofactor S-adenosylmethionine (SAM) to lysine and arginine residues of histone tails, respectively (see table 1, panel c). Histone methylation has no effect on DNA/histone interactions, but serves as a recognition template for effector proteins modifying the chromatin environment. Lysine methylation has been linked to both transcriptional activation and repression, as well as to DNA damage responses. In general, methylation at histone residues H3K4 and H3K36, including di-and trimethylation at these sites, is linked to actively transcribed genes, whereas H3K9 and H3K27 promoter methylation is considered as a repressive mark associated with heterochromatin [177]. However, methylation at different lysine residues, different degrees of methylation at the same lysine residue, as well as the locations of the methylated histones within a specific gene locus, may affect the functional consequences of these modifications. Histone methyltransferases (HMTs) have been classified according to their target (lysine or arginine) (table 1, panel c). Among the lysine methyltransferase's group (HKMTs), a further classification has been operated based on the presence or absence, and the nature of the sequences surrounding the catalytic SET domain [178]. Currently, at least seven SET domain families have been characterized: Suv39, SET1, SET2, EZ, RIZ, SMYD and Suv4-20 [178]. Until recently, histone methylation was regarded as irreversible. However, two kinds of histone demethylases (HDMTs) have been identified: the LSD1 (lysine specific demethylase 1) family and the Jumonji C (JmjC) domain family [179], which reverse histone methylation with both lysine-site and methyl-state specificity (see table 1, panel d).

Studying the implication of these epigenetic marks in the establishment and maintenance of HIV-1 latency has opened new therapeutic perspectives for manipulating epigenetic control mechanisms in order to activate viral transcription in latently-infected cells. In the next parts of this review, we draw the current portrait of the epigenetic control of HIV-1 transcription and we underline the potential of some new pharmacological agents to address the purge of the latent reservoirs.

## Nucleosomal organization of the 5'LTR of HIV-1

Our laboratory has previously studied the chromatin structure of integrated HIV-1 proviruses in several latently-infected cell lines by nuclease digestion methods [72]. Independently of the site of integration, two nucleosomes, named nuc-0 and nuc-1, are precisely positioned in the 5'LTR in basal conditions, and delineate two large nucleosome-free regions of chromatin corresponding to the enhancer/promoter region (nt 200 to 465; HS2+3) and to a regulatory region located downstream of the transcription start site (called HS4 and encompassing nt 610 to 720) (Fig 2C)[120].

The silent proviral 5'LTR can be switched on from postintegration latency by cell treatment with a variety of stimuli, including cytokines (i.e. IL-6 and TNF-α), antibodies (anti-CD3) or phorbol esters (PMA, prostratin), and by the viral protein Tat. In order for the transcriptional machinery to gain access to DNA, the chromatin structure needs to be altered. The nucleosome nuc-1, located immediately downstream of the transcription start site, is specifically remodeled following PMA or TNF-α treatment of the cells, coinciding with activation of HIV-1 gene expression [72,73]. This remodeling includes posttranslational modifications of histone tails and alterations of the chromatin structure by ATP-dependent remodeling complexes, whose importance is described hereafter.

## HDACs and HATs recruitment: a switch from latent to active transcription

HIV-1 transcriptional activation was shown to occur following treatment with several HDAC inhibitors (HDACIs) such as Trichostatin A (TSA), Trapoxin (TPX), Valproic Acid (VPA) and sodium butyrate (NaBut) either in cells transiently or stably transfected with HIV-1 LTR promoter reporter constructs [97,180,181], or using *in vitro *chromatin reconstituted HIV-1 templates [182,183], or in latently-infected cell lines [73], or in *de novo *infections [184]. These results indicate that nuc-1 is constitutively deacetylated by HDACs in latent conditions. The HDACI-mediated transcriptional activation is accompanied by the specific remodeling of nuc-1 and by an increased acetylation of H3K4 and H4K4 (activating epigenetic marks) in the promoter region [111,185].

Several transcription factors binding to the 5'LTR were demonstrated to recruit HDAC-1 (Fig 3A), whose inhibition promotes effective RNAPII binding to the HIV-1 promoter region, thereby allowing transcriptional initiation. A non exhaustive description of transcription factors which could be implicated in HDACs recruitment is described below with possible approaches to hinder recruitment (Figure 3A):

- LSF (Late SV40 Factor) binds to the 5'LTR downstream of the transcription start site and recruits YY1 (Ying Yang 1) via a specific interaction with its zinc-finger domain; YY1 subsequently recruits HDAC-1 [186,187]. Interestingly, pyrole-imidazole polyamides are small DNA-binding molecules which are specifically targeted to LSF binding sites and block the recruitment of HDACs to the HIV-1 5'LTR [185], leading to a transcriptional activation of HIV-1 in latently-infected cells [188].

- The unliganded form of thyroid hormone receptor (TR) decreases local histone acetylation following HDAC recruitment, while thyroid hormone treatment reverses this effect by nuc-1 remodeling and transcriptional activation [189,190].

- AP-4 (Activating Protein-4) represses HIV-1 gene expression by recruiting HDAC-1 as well as by masking TBP (TATA-binding protein) to the TATA box. This transcription factor is present concomitantly with HDAC-1 at the 5'LTR in latently-infected cells and dissociates following TNF-α activation as shown by chromatin immunoprecipitation (ChIP) assays [191].

- As described above, NF-κB p50/p50 homodimers recruit HDAC-1 to repress HIV-1 transcription in latently-infected cells.

- CBF-1 (C-promoter Binding Factor-1) binds to two sites embedded within the NF-κB/NFAT enhancer element. Knock-down of this factor causes an elevated H3K4 acetylation level and inhibits HDAC-1 recruitment to the 5'LTR [192].

- Stojanova *et al*. [193] have shown that the ectopic expression of c-Myc inhibits HIV-1 gene expression and virus production in CD4^+ ^T lymphocytes. This repression could involve c-Myc interaction with the initiator binding proteins YY1 and LBP-1 (Lipopolysaccharide-Binding Protein 1) [193] or c-Myc mediated recruitment of DNMT3A (DNA methyltransferase 3A) to the HIV-1 promoter [194]. Moreover, another group demonstrated that c-Myc is recruited to the HIV-1 5'LTR by Sp1 and in turn recruits HDAC-1 in order to blunt HIV-1 promoter expression [195]. Interestingly, small-molecule reagents that inhibit c-Myc have entered early clinical testing in oncology [196].

- RBF-2 (Ras-responsive Binding Factor 2) is composed of a USF-1/USF-2 (Upstream Stimulatory Factor) heterodimer whose cooperative association with the transcription factor TFII-I allows binding to the highly conserved upstream element RBEIII in the HIV-1 5'LTR [197,198]. HDAC-3 was demonstrated to modulate some of the functions of TFII-I [199] and RBEIII site mutation to inhibit HDAC-3 association with the 5'LTR of HIV-1 [200]. Moreover, the presence of HDAC-3 *in vivo *in the HIV-1 5'LTR region has been demonstrated in Jurkat J89 GFP cells [201]. These results suggest an implication of RBF-2 in the recruitment of HDAC-3 to the HIV-1 5'LTR but need further investigation.

- Sp1 binds to three sites immediately upstream of the core promoter and recruits HDAC-1 and HDAC-2 to promote histone H3 and H4 deacetylation [202,203]. In microglial cells, the CNS-resident macrophages, this recruitment requires the cofactor CTIP-2 (COUP-TF interacting protein 2), as described later in this review.

All these mechanisms are not mutually exclusive, and they highlight a unique redundant use of cellular transcription factors by HIV-1 to maintain quiescence in resting T cells. These mechanisms depict the complexity of this lentivirus' transcriptional regulation. Moreover, recent studies suggest a cooperative role in HIV-1 silencing of HDAC-1, HDAC-2 and HDAC-3, which could functionally substitute for each other [201,204]. Therefore, these redundancy properties could represent a way for the virus to ensure its replication in various cellular environments.

Following activation, cellular HATs, including p300/CBP, PCAF and Gcn5, are recruited to the promoter region leading to the acetylation of both H3 and H4 histones [111,202]. Several transcription factors have been shown to interact with HATs (Figure 3B), including AP-1, cMyb, GR, C/EBP, NFAT [121], Ets-1 [205], LEF-1 [206], NF-κB p50/p65 heterodimer [114], Sp1, IRF [207] and the viral protein Tat [208]. Furthermore, the ATPase subunit of SWI/SNF is recruited to the 3' boundary of nuc-1 by ATF-3, which binds to the second AP-1 site identified in the HS4 region, following PMA-mediated activation of Jurkat T cells [209] and/or by the viral protein Tat [209-212] as described in details here below. The maintenance of a stable association between the SWI/SNF subunit BRG-1 and chromatin appears to be dependent upon histone acetylation [209].

By altering histones, recruiting other chromatin-remodeling factors and modifying the activity of certain transcription factors, HDACs (and particularly HDAC-1) appear to be critical for the epigenetic repression of HIV-1 transcription and for the maintenance of latency. Following recruitment of HATs and chromatin remodeling complexes, nuc-1 disruption allows viral transcriptional activation to occur.

## HDAC inhibitors: near the cure?

We have previously reported that treatment of latently HIV-1-infected cell lines with HDACIs induces viral transcription and the remodeling of the repressive nucleosome nuc-1 [73]. HDAC inhibitors can be classified into five structural families: short-chain fatty acids (VPA, NaBut, phenylbutyrate), hydroxamates (TSA, suberoylanilide hydroxamic acid or SAHA, Scriptaid), benzamides (MS-275, CI-994), electrophilic ketones (trifluoromethylketone) and cyclic tetrapeptides (TPX, apicidin, depsipeptide) [213-215]. They act with varying efficiency and selectivity on the four different classes of HDACs and even between the different members of a same HDAC class [216,217]. In the case of HIV-1, potent inhibitors specific for class I HDACs might be effective therapeutics to disrupt latent infection and avoid toxicities that could accompany the global inhibition of members of the other HDAC families.

HDACIs present several advantages as a potential inductive adjuvant therapy in association with efficient HAART to purge latent reservoirs [143,218,219]. They activate a wide range of HIV-1 subtypes [184] without the toxicity associated with mass T-cell activation, which would generate new target cells for neo-synthesized viruses. HDACIs have even been demonstrated to repress the coreceptor CXCR4 in a dose-dependent manner [220]. They act on a broad spectrum of cell types; and therefore, in contrast to agents that specifically induce T cells, they could target the different latent reservoirs (macrophages, dendritic cells and other non-T cells). The most important element regarding the therapeutic goal resides in the fact that HDACIs have been safely administered to patients for several years in other human diseases: phenylbutyrate in β-chain hemoglobinopathies such as β-thalassemia and sickle cell anemia [221,222] and VPA in epilepsy and bipolar disorders [223,224]. More recently, SAHA (marketed as Vorinostat) was approved by the Food and Drug Administration (FDA) for treatment of cutaneous T-cell lymphoma [225]. In the context of many tumor cells, inhibitors of HDACs have been found to cause growth arrest, differentiation and/or apoptosis, but to display limited toxicity in normal cells [226]. Several HDACIs are engaged in various stages of drug development, including clinical trials for evaluation of their anti-cancer efficacy [215].

HDACIs also present certain limitations. General effects of HDAC inhibition on gene transcription should be a barrier to their wide clinical use. Various studies using cDNA arrays have shown that between 2% and 20% of cellular expressed genes are altered in cells exposed to HDACIs [215,217,227]. These genes are either activated or repressed. In addition, numerous non-histone proteins can be modified by acetylation and, depending on the functional domain involved, acetylation can alter different properties of these proteins such as DNA recognition, subcellular localization, protein-protein interactions and protein stability and/or activity. Therefore, inhibition of HDAC activity affects various biological processes [228,229]. Moreover, DNA-hypermethylation and subsequent compact heterochromatin formation may block the access of acetylases to their targets, thereby inducing resistance to HDACIs. Therapeutics that do not directly inhibit HDACs but that prevent their occupancy or action at the HIV-1 5'LTR may be considered as an alternative or an additional approach.

In 1996, VPA was shown to induce HIV-1 expression *in vitro *in latently-infected cells [230]. The Margolis group reported that VPA, in the presence of IL-2, provokes rescue of replication-competent HIV-1 from purified resting CD4^+ ^T cells obtained from HAART-treated patients with undetectable viral load [231]. Next, the same group evaluated the ability of clinically tolerable doses of VPA to deplete HIV-1 infection in a small clinical trial including four patients. To prevent the spread of infection during VPA treatment, they intensified HAART with enfuvirtide, a peptidic fusion inhibitor. After three months of treatment, they observed a modest but significant decline of the latent reservoir size in three of the four patients [232]. Later reports have failed to show a decay of infected resting CD4^+ ^T-cell latent reservoir following VPA treatment [233-236]. More specifically, two of these studies casted doubt on the effect of VPA, attributing the observed decline to HAART intensification with enfuvirtide because they failed to demonstrate a decline of the HIV-1 reservoir following VPA treatment [233,234]. The other two studies examining HIV-1-infected patients receiving VPA for neurologic purposes showed either a rapid rebound of plasma viremia even after two years of treatment [235] or could not observe a decrease in the size of reservoirs [236]. As VPA is a weak HDACI, other more potent and selective HDACIs were explored as therapeutic tools. The FDA-approved SAHA, a HDACI selective for class I HDACs, was shown to induce HIV-1 transcription in cell line models of postintegration latency and in CD4^+ ^resting T cells from aviremic patients under HAART [204,237]. In the Jurkat J89 GFP cell model, Archin *et al*. reported a decreased HDAC-1 occupancy at the 5'LTR and a concomitant nuc-1 acetylation following SAHA treatment of the cells [204]. Exposures to SAHA did not upregulate surface activation markers or receptors required for HIV-1 infection in PBMCs, which constitute good properties to avoid *de novo *infection. SAHA is thus a promising candidate for eradication of HIV-1-latent reservoirs and is under further investigation.

In conclusion, in strategies aimed at purging HIV-1 cellular reservoirs, HDACIs represent a potentially promising group of pharmacological agents. Among their numerous advantages, they activate HIV-1 transcription in postintegration latency model cell lines and in PBMCs from HAART-treated patients. However, at the present time, studies performed with these compounds, when used alone, have not reached the expected therapeutic goal, i.e. the eradication of latent reservoirs in HIV-1-infected patients.

## The viral Tat protein and Tat-associated factors

HIV-1 transcription is characterized by an early Tat-independent phase, where the promoter is under the control of the chromatin environment and cellular host transcription factors. This phase is followed by a late Tat-dependent phase, where Tat primarily drives high levels of transcription (reviewed in [238] and [239]).

**Figure 4:**
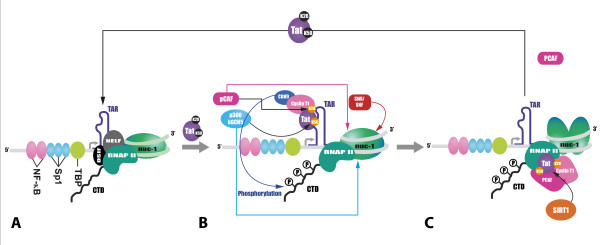
**Mechanisms of transcriptional activation by the viral protein Tat**. (A) In the absence of Tat, transcription from the HIV-1 5'LTR produces predominantly short mRNAs as a result of the activity of the negative elongation factor N-TEF, composed of NELF and DSIF, which binds to the hypophosphorylated RNA polymerase II and impedes transcriptional elongation. (B) Following the synthesis of the first molecules of Tat, this viral protein migrates to the nucleus. Tat then binds to the RNA hairpin TAR, located in the 5' region of all nascent HIV-1 transcripts and activates viral transcription by recruiting the positive elongation factor pTEFb, composed of Cdk9 and CyclT1. This recruitment is enhanced through Tat acetylation by PCAF on K28, located in the transactivation domain of the viral Tat protein. Cdk9 phosphorylates the CTD domain of RNAPII, leading to processive transcriptional elongation and to the dissociation of N-TEF. Acetylation of Tat on K50 by p300 and Gcn5 promotes the release of pTEFb [246], dissociation of Tat from TAR and its subsequent transfer to the elongating polymerase complex. Tat then recruits PCAF to the elongation complex. Tat also recruits the ATP-dependent remodeling complex SWI/SNF. Another model based on FRAP experiments propose that the Tat/pTEFb complex dissociates from the RNAPII complex following transcription initiation and undergoes subsequent cycles of association/dissociation [249]. (C) At the end of the elongation process, Tat deacetylation by the class III HDAC Sirtuin 1 allows its dissociation from RNAPII and from PCAF, and the recycling of Tat initiates a new cycle of transcriptional activation.

In the absence of Tat, transcription is initiated but blocked at the promoter proximal position in an early elongation stage (see Fig 4). This block is due to the presence of N-TEF (negative transcription elongation factor), composed of two subunits NELF (negative elongation factor) and DSIF (DRB-sensitive inducing factor) [240,241], as well as to a repressive chromatin environment. Short abortive transcripts of about 60 nt in length accumulate in the cytoplasm [242], but occasional full-length viral genomic transcripts would allow the synthesis of a few molecules of Tat sufficient to stimulate HIV-1 transcription. Tat binds to the stem-loop TAR RNA element present at the 5'end of all nascent viral transcripts and recruits to its N-terminal domain the factor pTEFb (positive transcription elongation factor b), composed of the Cyclin T1 and of the kinase Cdk9 (Fig 4B). This recruitment is enhanced through Tat acetylation by PCAF on K28, located in the trans-activation domain of the viral protein [180]. Cdk9 phosphorylates the CTD of RNAPII, promoting efficient elongation [238,243]. In the presence of Tat, the substrate specificity of Cdk9 is altered, such that the kinase phosphorylates both serine 2 and serine 5 of the CTD instead of serine 2 alone [244]. In addition, N-TEF phosphorylation mediated by pTEFb relieves the block to transcriptional elongation [245]. Tat itself is also acetylated on K50 by p300 and Gcn5 in order to promote the release of pTEFb [246], dissociation of Tat from TAR, and its subsequent transfer to the elongating RNAPII complex. Tat can then recruit PCAF [247,248], which could play a role in chromatin remodeling following transcriptional activation. Of note, FRAP experiments showed that the Tat/pTEFb complex dissociates from the RNAPII complex following transcription initiation and undergoes subsequent cycles of association/dissociation [249]. At the end of the elongation process, Tat deacetylation by sirtuin 1 (SIRT1), a class III protein deacetylase, allows its dissociation from the RNAPII and PCAF complex, and its recycling to initiate a new cycle of transcriptional activation [250] (Fig 4C). This function might be important when the amount of Tat is a limited factor, especially at the early phase of infection.

The binding of Tat to TAR also promotes the recruitment of various cellular cofactors to the HIV-1 5'LTR including histone-modifying enzymes such as the HATs p300 and CBP [208] and chromatin remodeling complexes [209-212,251], likely reinforcing an acetylated and open chromatin environment [251]. Tat first recruits the ATP-dependent remodeling complex SWI/SNF via its interaction with BRG-1 and Ini1 subunits, allowing the initiation of nuc-1 remodeling [212]. Acetylated Tat on K50 interacts with the subunits BRM and Ini1 of another SWI/SNF complex [211], which is consequently recruited at the 3' end of nuc-1 in the 5'LTR and completes nuc-1 remodeling to facilitate transcriptional elongation [252]. The histone chaperone hNAP1 also interacts with Tat improving its stability and increasing the level of chromatin folding, probably in cooperation with p300 [253]. In addition to acetylation, Tat itself is subject to other posttranslational modifications. Indeed, this viral protein can be methylated by the protein arginine methyltransferases PRMT6 on its R52 and R53 residues, resulting in a decreased interaction with TAR and counteracting pTEFb complex formation [254,255]. One or several protein lysine methyltransferases, at least SETDB1, were demonstrated to methylate Tat on K50 and K51 residues [256], thereby competing with acetylation of the same residues. SETDB1 was shown to recruit DNMT3a and HDACs in order to promote gene silencing and heterochromatin formation [257,258]. Together, these results suggest that Tat acetylation is associated with active transcription, whereas Tat methylation mainly interferes with transcription and promotes HIV-1 latency.

Beside its classically recognized role in induction of transcriptional elongation and chromatin remodeling, Tat may also influence transcriptional initiation by facilitating assembly of the pre-initiation complex [239] requiring the Sp1 and NF-κB binding sites, but no consensus about the mechanisms involved has been reached so far [259-261]. Increasing evidence suggests that Tat also plays a role in splicing, capping and polyadenylation processes [262,263].

In conclusion, Tat acts at several levels in HIV-1 transcription. Weinberger and colleagues showed that Tat level fluctuation is a crucial event that may influence the switch from a lytic productive state of the infection to a latent non-productive state [264].

Beside low levels of Tat, latency might also result from low levels of Tat-associated factors, such as CycT1/Cdk9. Expression of pTEFb is activated by cytokines IL-2 and IL-6 [265]. The kinase activity of the complex CycT1/Cdk9 is constitutively restricted by its association with a small cellular RNA named 7SK, which acts as a scaffold for HEXIM1, a cellular protein containing a C-terminal pTEFb inhibitory domain [266]. Hexamethylene bisacetamide (HMBA) is a clinically tolerable agent [267], first developed as an anticancer drug, which could be of interest in the reactivation of the latent reservoirs. HMBA causes the release of pTEFb from HEXIM1 and triggers Cdk9 recruitment to the HIV-1 5'LTR via an unexpected interaction with the transcription factor Sp1 [268]. HMBA was shown to induce gene expression in latently-infected T-lymphoid and monocytic cell lines, and to provoke a downregulation of the receptor CD4 but not of the coreceptors CXCR4/CCR5 at the PBMC surface [269]. Pilot human clinical trials suggest that HMBA, or other analog compounds, might be developed as therapeutics to target HIV-1 latently-infected cells.

## Histone methylation status and heterochromatin environment of the HIV-1 integrated promoter

**Figure 5:**
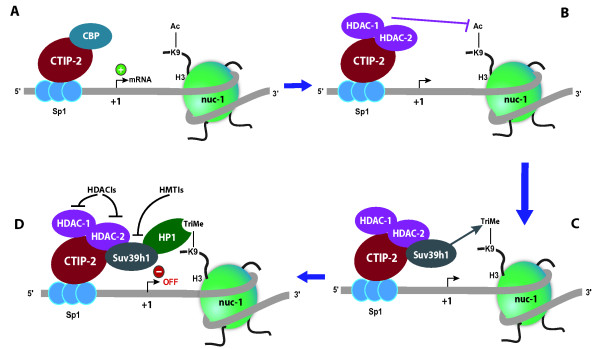
**Model for CTIP-2-mediated establishment of a heterochromatin environment at the HIV-1 promoter region**. (A) CBP recruitment occurs following HIV-1 activation with phobol esters via CTIP-2, which binds to Sp1 binding sites. (B) However, in latent conditions, the corepressor CTIP-2 interacts with the Sp1 transcription factor at three sites in the HIV-1 5'LTR and consequently recruits HDAC-1 and HDAC-2, leading to H3K9 deacetylation in the nuc-1 region. (C) CTIP-2 then recruits the HMT Suv39 h1, which trimethylates H3K9 (a repressive chromatin mark). (D) This latter epigenetic modification allows HP1 binding and polymerization, heterochromatin formation and propagation at least to the nuc-2 region, and *in fine *the establishment of HIV-1 silencing [202,203]. This mechanism of viral latency can be revoked by different treatment strategies: HDACIs hinder the repressive action of HDACs and specific HMTIs directed against Suv39 h1 could avoid the recruitment of this silencing machinery.

In addition to histone hypoacetylation, H3K9 trimethylation is associated with a repressive chromatin status [270]. This epigenetic mark is mediated by the human histone methyltransferase Suv39 h1. The subsequent recruitment of HP1 initiates heterochromatin formation, whereas propagation and maintenance of heterochromatin are guaranteed via a self-perpetuating epigenetic cycle involving HP1, Suv39 h1 and H3K9 trimethylation [271]. Interestingly, recruitment of these factors at the HIV-1 5'LTR has been previously described in microglial cells [203]. Indeed, the group of Rohr in collaboration with our laboratory has demonstrated that the transcription factor Sp1, which binds to its three cognate binding sites in the 5'LTR, recruits a multienzymatic chromatin-modifying complex to the HIV-1 promoter via the transcriptional corepressor CTIP-2 (Fig 5) [202]. This corepressor was initially identified in association with members of the COUP-TF family [272]. CTIP-2 is mainly expressed in the brain and the immune system [273]. Concomitant recruitment of HDAC-1, HDAC-2 and Suv39 h1 to the viral promoter by CTIP-2 allows H3K9 deacetylation, which is a prerequisite for H3K9 trimethylation by Suv39 h1 [203] (Fig 5). This last histone modification allows HP1 binding and polymerization. Moreover, CTIP-2 itself can recruit Suv39 h1 and the three isoforms HP1α, HP1β and HP1γ of HP1 proteins to the viral promoter [274]. Chromatin modifications (e.g. H3K9 deacetylation and further H3K9 trimethylation) appear to be propagated in the downstream nuc-2 region, suggesting that this heterochromatic structure spreads along the viral genome [202]. Interestingly, the Rohr group reported displacement of CTIP-2 and subsequent recruitment of CBP through Sp1 following HIV-1 activation with phobol esters [202]. Moreover, the Benkirane's group has demonstrated that Suv39 h1, H3K9 trimethylation and HP1γ play a major role in chromatin-mediated repression of HIV-1 gene expression in systems other than microglial cells, including HIV-1 infected T-cell lines and PBMCs from infected individuals [275]. Indeed, these authors demonstrated an increased level of HIV-1 expression in cultures of primary HIV-1-infected CD4^+ ^T cells following siRNA knockdown of Suv39 h1 and HP1γ proteins [275].

In addition to repressing the initial phase of HIV-1 transcription through direct binding to the 5'LTR via Sp1, CTIP-2 also specifically acts as a potent inhibitor of Tat-mediated transcriptional activation [274]. By direct interaction with Tat N-terminal domain and in association with a third partner HP1α, CTIP-2 leads to the relocalization of this tripartite protein complex Tat-CTIP2-HP1α in distinct nuclear regions associated with heterochromatin [274]. These observations strengthen the hypothesis that the formation of nuclear bodies inhibits HIV-1 gene transcription by sequestering a variety of factors required for transcriptional activation [276]. Of note, PML (promyelocytic leukemia) bodies are sub-nuclear compartments that have been shown by certain groups to inhibit HIV-1 through different mechanisms: (i) by modulating the availability of essential transcription cofactors such as CyclinT1 [277], thereby regulating Tat-mediated transcriptional trans-activation; (ii) by triggering the exportin-mediated cytoplasmic export of the Ini1 subunit of the SWI/SNF complex in association with PML proteins [278].

These epigenetic silencing elements further participate in the complexity of HIV-1 latency and represent potent targets for inducing HIV-1 gene expression in latently-infected cells. Beside HDACIs, histone methyltransferase inhibitors (HMTIs) represent new candidate drugs for purging the HIV-1 latent reservoirs. Despite their compelling interest in cancer therapy, the search for HMTIs is still at its beginning [279]. SAM-analogs such as methylthioadenosine, *S*-adenosylhomocysteine, or the bacterial metabolite sinefungin have been developed [280], but their lack of specificity will require further investigations to address their effect on host cell gene expression. Indeed, these compounds affect enzymes other than HMTs which use SAM as a cofactor, such as DNMTs (DNA methyltransferases). Three specific inhibitors of lysine methyltransferases have been described so far: chaetocin, which specifically inhibits Suv39 h1 [281], 3-deazaneplanocin A (DZNep) [282], and BIX-01294 acting on the G9a HMT (G9a targets H3K9 and H3K27) [283]. As Suv39 h1-mediated trimethylation requires the previous demethylation of H3K4 by LSD1 [284], histone demethylase inhibitors (HDMTIs), such as parnate, could also be of interest to activate HIV-1 latently-infected cells. Further studies aiming at identifying new HMTIs should provide additional compounds to be tested for their possible role in activation of latent HIV-1 reservoirs.

## DNA methylation in HIV-1 latency: an area of enhanced interest

CpG methylation of the HIV-1 promoter seems to be an important epigenetic mechanism that maintains latency as was previously reported for several retroviruses as HTLV-1 (Human T-cell leukemia virus) [285,286], and MMLV (Moloney Murine Leukemia Virus) [287]. Catalyzed by DNA methyltransferases (DNMTs) (Table 1, panel e), DNA methylation occurs predominantly at cytosine residues located within CpG dinucleotides and is associated with transcriptional silencing. DNA methylation may impact transcription in two ways [288]: (i) the methylated DNA may itself physically impede the binding of transcription factors to their binding site; (ii) methylated DNA may be bound by methyl-CpG-binding domain proteins (MBDs), which in turn interact with HMTs (such as Suv39 h1) and with HDACs, leading to a repressive chromatin structure. This link between DNA methylation and histone epigenetic marks is important for our understanding of the establishment of a latent infection.

CpG methylation of the HIV-1 5'LTR was first reported in stably-transfected fibroblasts [289]. At the functional level, CpG methylation of the HIV-1 promoter was shown to inhibit transcription in a CAT reporter assay with *in vitro *methylated vectors [290,291] and was suggested as a mechanism to maintain HIV-1 latency in U937 monocytic cells [292]. DNA methylation probably acts by impairing the binding of several important transcription factors to the 5'LTR, such as NF-κB, USF and Sp1 [290]. Various anticancer agents including 5-aza-2'deoxycytidine (5-Aza-CdR), an FDA-approved inhibitor of DNA methylation used in humans to treat myelodysplastic syndrome (marketed as Decitabine) [293], were shown to induce HIV-1 transcription in latently-infected cell lines [294] and in a doxycycline-dependent HIV-rtTA variant [99]. However the role of DNA methylation in HIV-1 latency was still controversial, and some laboratories even reported that CpG methylation did not correlate with transcriptional silencing [295].

Recently, two groups have independently pointed the importance of DNA methylation in HIV-1 provirus behavior and latency. Two CpG islands overlapping two regions in the 5'LTR previously shown to be nucleosome-free and enriched in transcription factor binding sites were demonstrated to be hypermethylated in J-Lat cells, a model cell line for postintegration latency. Methyl-binding domain protein 2 (MBD-2) and HDAC-2 were shown to be recruited to the latent HIV-1 promoter via the second CpG island located downstream of the transcription start site. MBD-2 might silence transcription by the recruitment of the NuRD complex, a hypothesis supported by the fact that HDAC-2 is part of the NuRD complex [296]. Furthermore, although 5'LTR cytosine methylation is not required to establish latency, latency controlled solely by transcriptional interference and chromatin-dependent mechanisms in absence of significant 5'LTR promoter DNA methylation tends to be leaky and easily reactivatable [297]. In contrast, CpG methylation of the 5'LTR, probably in concert with repressive histone modifications (such as low levels of H3K4 dimethylation and of H3 acetylation, and high levels of H3K9 trimethylation), contributes to "lock" the silent state of the provirus by preventing its reactivation [297]. Despite previous discrepancies about the link between DNA methylation in the 5'LTR and HIV-1 latency, the importance of this epigenetic modification is today well established and must be envisaged in strategies to purge latent reservoirs.

In addition to the four well-characterized nucleoside analog methylation inhibitors, 5-azacytidine (5-Aza), 5-aza-2'-deoxycytidine, 5-fluoro-2'-deoxycytidine, and zebularine, there is a growing list of non-nucleoside DNA methylation inhibitors such as procaine, procainamide, hydralazine and RG108 [298]. Only 5-Aza and 5-Aza-CdR are currently FDA-approved and used in cancer therapies. The major hindrance of their usage in humans is their instability *in vivo *and the toxicity secondary to their excessive incorporation into DNA, which causes cell cycle arrest. These cytosine analogs were also shown to induce proteasomal degradation of DNMT1 [299]. Today, no clinical study including HIV-1 infected patients or *ex vivo *reactivation studies on PBMCs isolated from HAART-treated patients have been performed using DNA methylation inhibitors to reduce the pool of latent reservoirs, but it is an interesting field to explore.

## Combination therapy approaches to purge HIV-1 reservoirs

Current HAART decreases HIV-1 RNA plasma levels below 50 copies/ml but still does not eradicate the virus from infected patients. Interrupting HAART leads to a rapid viral rebound attributed to the persistence of latently-infected cellular reservoirs. Latency is a multifactorial phenomenon: different levels of transcriptional and epigenetic blocks are involved and probably act in concert to silence HIV-1 transcription. Altogether DNA- and histone-methylation and histone deacetylation cooperate to establish and maintain a repressive chromatin structure of the HIV-1 provirus in latently-infected cells. Moreover, several studies in cells from aviremic HAART-treated patients have shown that targeting latent reservoirs with an HDACI alone or a PKC agonist alone could decrease the size of the latent pool, but inevitably led to a rapid viremia rebound in the several weeks following interruption of the treatment [233-236]. New strategies attacking simultaneously different levels of latency maintenance and/or establishment should be more efficient when viral eradication is the objective since the combination of different types of compounds could synergize in the reactivation of latently-infected cells.

So called "Shock and kill" strategies are based on activation of HIV-1 expression followed by stimuli leading to elimination of infected cells, either naturally (via the host immune system or viral cytopathic effects), or via a "kill" phase treatment (with drugs or antibodies). Savarino *et al*. have recently combined HDACIs as HIV-1 inducers and BSO (glutathione-synthesis inhibitor buthionine sulfoximine), which creates a pro-oxidant environment and in turn stimulates HIV-1 transcription [300]. BSO increased HDACIs ability to induce HIV-1 expression by lowering intracellular levels of glutathion, thereby allowing the reduction of both drug concentrations to doses that were not toxic for uninfected cells. Moreover, BSO induced the recruitment of HDACIs insensitive cells to the population of responding cells [300]. This combination thus potentiates the action of HDACIs, but different activation strategies may be required to reach each type of latent reservoirs.

Our laboratory has previously demonstrated a strong synergistic activation of the HIV-1 promoter activity by the combination of the HDACI TSA and the NF-κB inducer TNF-α in the postintegration latency promonocytic model cell line U1 [184,301]. It is interesting to note that an array of cytokines, including TNF-α and IL-1, is already copiously expressed in the environment of lymphoid tissues, and that they could amplify the clinical potential of HDACIs in patients. However, toxicity of these compounds (TNF-α and TSA) undermines their clinical interest for human therapy. Our laboratory has examined the HIV-1 reactivation potential of a treatment combining the non tumor-promoting NF-κB inducer prostratin and several HDACIs used in human clinical therapies (such as VPA and SAHA)[302]. This study demonstrated a synergistic activation of HIV-1 gene expression by these combinations of compounds in latently-infected cell lines (promonocytic U1 cells and J-Lat T cells). In this latter cell line, FACS experiments showed the recruitment of latently-infected cells to the population of HIV-1 actively expressing cells following the treatment HDACI+prostratin [302]. The observed synergistic activation was accompanied by the remodeling of the nucleosome nuc-1 as shown by indirect end-labeling experiments. As mentioned above, whereas two studies have suggested that VPA could be a good candidate to deplete latent infection [231,232], more recent studies did not confirm these results [233-236,302]. However, the inability of VPA to reactivate latent reservoirs when used alone showed potentiating results when used in combination with prostratin [302]. Therefore, when combined with other kinds of HIV-1 inducers, VPA could have an impact on the decay of latent reservoirs, despite its weak HDAC inhibitor activity. Finally, *ex vivo *cultured CD8^+^-depleted PBMCs isolated from the blood of aviremic HIV-1-infected patients under HAART and with undetectable viral load were treated with these combinations (HDACI+prostratin). HIV-1 expression was synergistically reactivated in 60% of the patients tested (25 out of 42 patients) [302]. These results constitute a proof-of-concept for the co-administration of (at least) two different categories of therapeutically promising HIV-1 inducers together with HAART in order to decrease the pool of latent HIV-1 reservoirs. However, 40% of the patient samples didn't show any viral outgrowth following these treatments. This could result from a stronger epigenetic control (including DNA and histone methylation) of the integrated provirus in those resting cells that would hinder viral transcription and reactivation.

Wu *et al*. showed that depsipeptide (an HDACI)-mediated activation of gene transcription was accompanied by a decrease of CpG methylation and H3K9 methylation in the promoter region [303]. These results pointed to a strong interplay between acetylation and methylation mechanisms. Moreover, as some latently-infected cells were reactivated by 5-Aza-CdR and not by TSA, demethylation seems to be a prerequisite to HDACI transcriptional activation in certain highly latent reservoirs [297]. Such combinations of a DNA methylation inhibitor and a HDACI are used in clinical trials as anticancer treatment [304]. A combination 5-Aza-CdR/VPA has been tested in latently-infected cell lines but failed to synergistically reactivate HIV-1 transcription [296]. However, in the same experiment, the authors showed that inhibiting provirus methylation leads to an almost complete reactivation of latent HIV-1 in J-Lat T cells when combined with the activator of NF-κB signaling TNF-α [296]. In another study, 5-Aza-CdR was also shown to synergize with prostratin, which triggers reactivation of latent HIV-1 without broad T-cell activation and inhibits *de novo *virus infection [141]. The additional use of DNA methylation inhibitors coupled with a PKC agonist or with a HDACI in antiretroviral therapy could be a further step to clear the virus from infected patients.

The study of the epigenetic mechanisms implicated in HIV-1 latency also indicates the involvement of histone methylation in the heterochromatin formation process in the 5'LTR region and suggests a probable effect of HMTIs and HDMTIs in combinatory strategies to eliminate HIV-1 reservoirs. Because differences in reactivation patterns were observed in all patient cell cultures tested, it is important to evaluate a broader panel of clinically available agents and to examine several combinations for each patient in order to reactivate all kinds of latently-infected cells and target the different mechanisms implicated in latency. Such combinatorial approaches, even if they don't achieve total eradication, could provide important HIV-1 activation leading to a decline of HIV-1 reservoir levels sufficient to allow for the efficient control of the infection by the host immune system, and allow individuals to envisage therapeutic interruptions.

## Concluding remarks

HIV-1 latent reservoirs are established early during primary infection and constitute a major obstacle to virus eradication. Understanding the complexity of the mechanisms involved in HIV-1 latency and the numerous links between different control levels of latency requires molecular approaches to determine the most potent targets to counteract repressive chromatin maintenance and gene silencing. In a therapeutic goal, the ideal compounds should be orally available, active but not toxic in a wide variety of cell types in order to reach HIV-1 sanctuaries such as the central nervous system and compatible with the different components of HAART. Since the discovery of latent reservoirs in the late nineties, the study of HIV-1 has followed a path strewn with obstacles, and its eradication is still far away. However, the growing understanding of the molecular mechanisms involved in this disease, and of the virus' unusual talent to escape treatments and its ability to establish and maintain a latent state in a wide variety of cells, allows us hope for a therapeutic breakthrough. Today, the most promising strategy to eradicate latent reservoirs resides in combinations of several families of compounds to force HIV-1 gene expression simultaneously at different levels.

## List of abbreviations used

5-Aza: 5-azacytidine; 5-Aza-CdR: 5-aza-2'-deoxycytidine; AP-1: Activating Protein-1; APOBEC3G: APOlipoprotein BmRNA Editing Catalytic subunit-like protein 3G; ATF-3: Activating Transcription Factor-3; ATP: Adenosine TriPhosphate; Bcl-2: B-cell Lymphoma 2; BRG-1: Brahma-Related Gene 1; BRM: Brahma Gene; BSO: buthionine sulfoximine; CAT: Chloramphenicol AcetylTransferase; CBF-1: C-promoter Binding Factor-1; CCR5: Chemokine CC motif Receptor 5; CD4: Cluster Designation 4; Cdk9: Cyclin-Dependent Kinase 9; ChIP: Chromatin ImmunoPrecipitation assays; C/EBP: CCAAT/enhancer binding protein family; c-Myc: v-myc myelocytomatosis viral oncogene homolog; CNS: Central Nervous System; COUP-TF: Chicken Ovalbumin Upstream Promoter-Transcription Factor; CTD: Carboxy-Terminal Domain; CTIP-2: COUP-TF Interacting Protein 2; CXCR4: Chemokine CXCMotif Receptor 4; CycT1: Cycline T1; DNA: DeoxyriboNucleic Acid; DNMT: DNA MethylTransferase; DNMTI: DNA MethylTransferase Inhibitor; dNTP: DeoxyNucleotide TriPhosphate; DPP: 12-DeoxyPhorbol 13-Phenylacetate; DSIF: DRB-Sensitive Inducing Factor; ERK: Extracellular signal-Related Kinase; FDA: Food and Drug Administration; FDC: Follicular Dendritic Cell; FRAP: Fluorescence Recovery After Photobleaching; GNAT: Gcn5-related N-AcetylTransferase; GFP: Green Fluorescent Protein; HAART: Highly Active AntiRetroviral Therapy; HAT: Histone AcetylTransferase; HATI: Histone AcetylTransferase Inhibitor; HDAC: Histone DeACetylase; HDACI: Histone DeACetylase Inhibitor; HDMT: Histone DeMethylase; HDMTI: Histone DeMethylase Inhibitor; HEXIM1: HMBA Inducible protein 1; HIV-1: Human Immunodeficiency Virus type 1; HMBA: HexaMethylene BisAcetamide; HMT: Histone MethylTransferase; HMTI: Histone MethylTransferase Inhibitor; HP1: Heterochromatin Protein 1; HS: Hypersensitive Site; HTLV-1: Human T-cell Leukemia Virus 1; IAT: Immune Activation Therapy; IKB: Inhibitor of NF-κB; IKK: IKappaB Kinase; IL-2: InterLeukin 2; Ini1: Integrase-interacting protein 1; IRF: Interferon (IFN)-Responsive Factor; JNK: c-Jun N-terminal Kinase; JmjC: Jumonji C; LBP-1: Lipopolysaccharide-Binding Protein 1; LEDGF: Lens Epithelial Derived Growth Factor; LEF-1: Lymphoid Enhancer Factor 1; LSD1: Lysine Specific Demethylase 1; LSF: Late SV40 Factor; LTR: Long Terminal Repeat; MBD: Methyl-CpG Binding Domain Protein; MeCP2: Methyl-CpG Binding Protein 2; MMLV: Moloney Murine Leukemia Virus; NaBut: Sodium Butyrate; Nef: Negative Regulatory Factor; NELF: Negative ELongation Factor; NFAT: Nuclear Factor of Activated T cells; NF-κB: Nuclear Factor Kappa B; N-TEF: Negative Transcription Elongation Factor; NuRD: Nucleosome Remodeling and Deacetylation; Oct1: Octamer-binding transcription factor 1; OKT3: Orthoclone K T-cell Receptor 3 antibody; PBMC: Peripheral Blood Mononuclear Cell; PCAF: p300/CBP-Associated Factor; PIC: Preintegration Complex; PKC: Protein Kinase C; PMA: Phorbol 12-Myristate 13-Acetate; PML: ProMyelocytic Leukemia; PRMT: Protein arginine MethylTransferase; pTEFb: Positive Transcription Elongation Factor b; PU.1: PU-box binding factor 1; RBF-2: Ras-responsive Binding Factor 2; RPD3: Reduced Potassium Dependency 3; RNAi: RNA interference; RT-PCR: Reverse Transcriptase-Polymerase Chain Reaction; SAM: S-AdenosylMethionine; SAHA: SuberoylAnilide Hydroxamic Acid; SET: Su(var)3-9 Enhancer-of-zeste and Trithorax; Sirt: (Sirtuin) Silent mating tape Information Regulation 2 homolog; Sp1: SV40-promoter specific factor;Suv39 h1: Suppressor of Variegation 3-9 Homolog 1; SWI/SNF: SWItching/Sucrose Non Fermenting; TAR: Tat Responsive element; Tat: TransActivator of Transcription; TCR: T-Cell Receptor; TNF-α: Tumor Necrosis Factor-α; TPX: Trapoxin; TR: Thyroid hormone Receptor; TRIM5α: Tripartite Motif protein 5 α; TSA: TrichoStatin A; USF: Upstream Stimulatory Factor; Vif: Viral Infectivity Factor; VPA: Valproic Acid; Vpu: Viral Protein U; YY1: Ying Yang Protein 1.

## Competing interests

The authors declare that they have no competing interests.

## Authors' contributions

LC and CVL contributed to the writing of the manuscript.
